# Supplementary material for: Gap junction-mediated contraction of myoepithelial cells induces the peristaltic transport of sweat in human eccrine glands
Source: Commun Biol. 2023 Nov 18;6:1175. doi: 10.1038/s42003-023-05557-9 (PMC10657463; doi:10.1038/s42003-023-05557-9)
Supplement: Supplementary file 2 — Supplementary Information [file 42003_2023_5557_MOESM2_ESM.pdf]

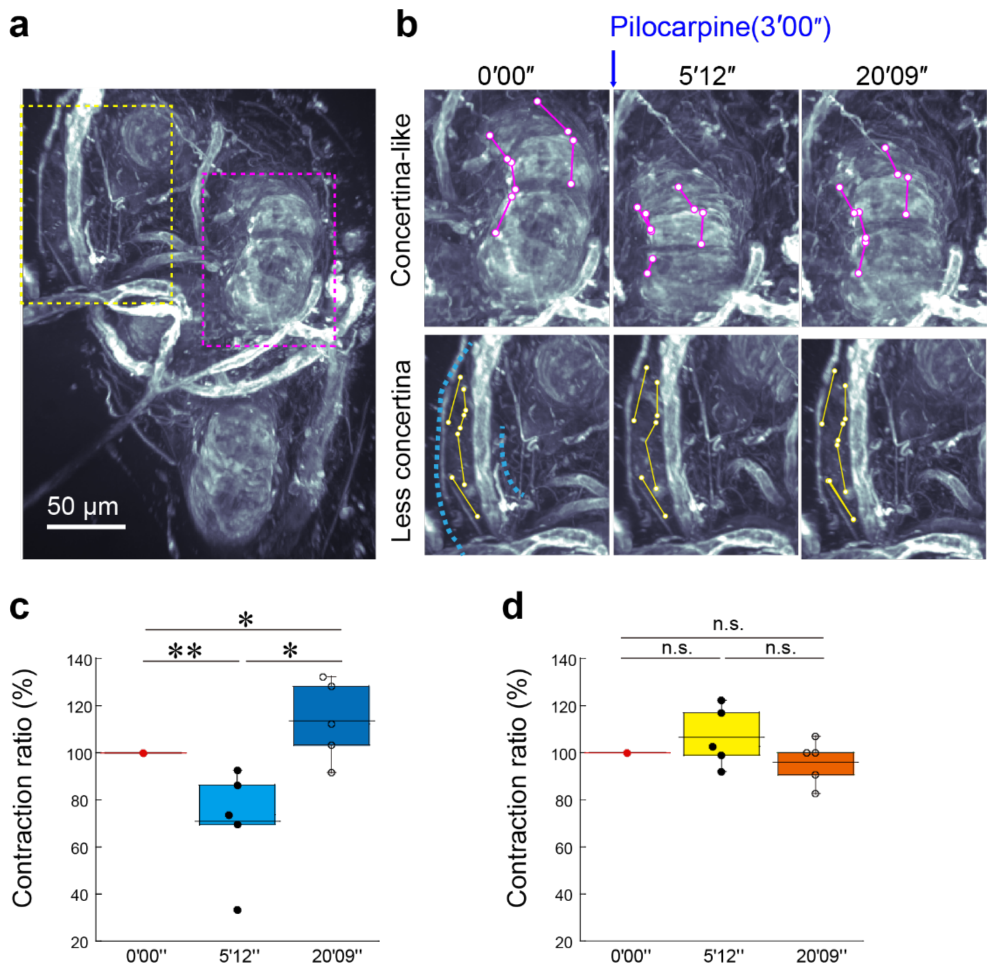

Supplementary Figure 1 (Figure S1)

Quantification of pilocarpine-induced contractile motion of the secretory duct. Pilocarpine-induced contraction of the secretory duct was assessed by measuring the distance between two virtual reference points selected on the concertina-like and less concertina-like regions of the secretory duct shown in **Supplementary movie S5.** Pilocarpine was added three minutes after the start of the observation period. a Low-magnification view of the entire CMDR-stained secretory duct (see also **Supplementary movie S5**). Boxed with dotted lines are the regions showing concertina-like (magenta) and less concertina-like (yellow) signals. **b** Snapshot views of the concertina-like (**upper panels**) and less concertina-like (**lower panels**) regions of the pilocarpine-treated secretory duct at 0'00" (**left**), 5'12" (**middle**), and 20'09" (**right**). Maximal contraction was observed at 5'12". Virtual reference points selected for quantification of the contractile motion of the secretory duct are shown as pairs of dots connected by lines. **c, d** Quantification of the distance between each pair of virtual reference points in the concertina-like (**c**; n=5) and less concertina-like (**d**; n=5) regions of the secretory duct. The contractile motions are expressed as the ratio of the distance at each time point to the distance at 0’00”. **Statistics**: unpaired t tests; *P<0.05, **P<0.01.

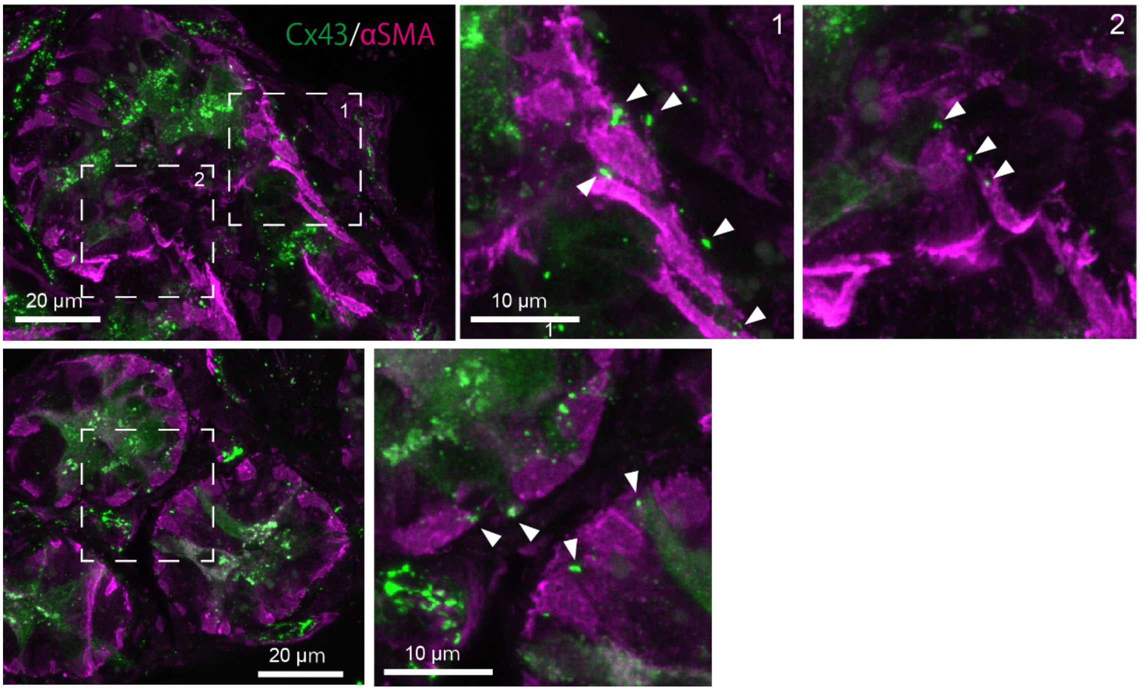

Supplementary Figure 2 (Figure S2)

High-magnification immunofluorescence images of Cx43 (green) costained with αSMA (magenta) in the secretory duct. Puncta of Cx43 signal were detected between myoepithelial cells in the secretory duct (white arrowheads). Magnified views of the boxed areas are shown to the right.

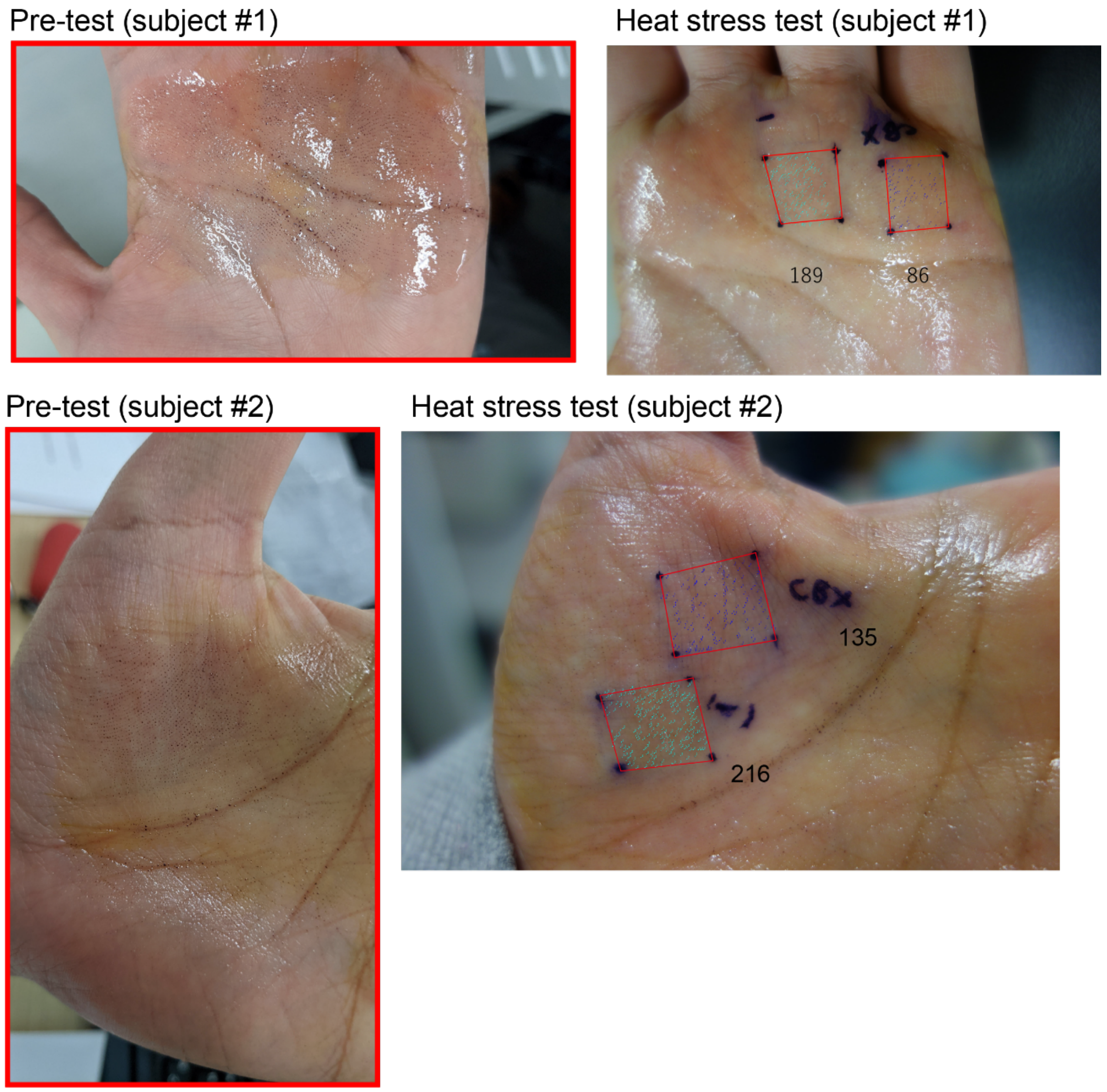

Supplementary Figure 3 (Figure S3)

Representative results of in vivo sweating tests. Prior to the heat stress test, active sweat pores were stained by the starch-iodine method (left panels boxed in red; "Pretest"). Right panels show the results of the heat stress test of the same subjects with and without CBX treatment. The areas exposed to the CBX-soaked (CBX) and PBS-soaked (Control) cotton pads are bounded by four dots.

Supplementary Table 1

Primers for qRT–PCR.

	Fw	Rev
Cx26	TCTTTTCCAGAGCAAACCGC	GACACGAAGATCAGCTGCAG
Cx30	AGGCACTCCAGTGGGGTAGGA	GTGCAGCGTCCCCCAATCCA
Cx30.3	TACCCACCTGCATCCACTGG	GGTGGACGTACTTGCTGAGC
Cx31	AATTCTCGCAGGTAGGCAC	CCAGAGAGTGTGCAGCAGGT
Cx31.1	GTGGACATATGTCTGCAGCC	CTATGAGAGATGCTAGAGC
Cx31.9	GCTGCTACCTGCTGAGCGT	TTCTGCGCCTCTTCGTGT
Cx32	GACAGGTTTGTACACCTTGC	CGTCGCACTTGACCAGCCGC
Cx37	GTTGCTGGACCAGGTCCAGG	GGATGCGCAGGCCACCATCT
Cx40	AGCGTGGGCAGTTGGAGAAGA	TGCCTACCACGGTCGAGTGCT
Cx40.1	TTCAGCGTCTATGTCCTGCA	GGAGGAGGAGGTGGATGATG
Cx43	AATTCAGACAAGGCCCACAG	CATGGCTTGATTCCCTGACT
Cx45	GGAGCTTTCTGACTCGCCTG	CGGCCATCATGCTTAGGTTT
Cx46	GGGCTACCAAGAGACACTGC	ACCTTCTCCTGCTCCTCCAT
Cx47	AGAGAGGCCTACGAGCCAGA	CAAGCTGCAAGGCATATCAA
Cx50	TCCACTCCATTGCTGTCTC	GTGGTCAGCCTCTCTGCTTC
Cx58	GAAATGCCTAGGGATCGGA	AACCTAGGTGGAAAATTTCAAGA
Cx62	AAGGATGTCTGCTGCGTACTTA	GCCTGTTTCATCCTCAATGC
18S	ACCCGTTGAACCCCATTCGTGA	GCCTCACTAAACCATCCAATCGG
