# Supplementary material for: Gap junction-mediated contraction of myoepithelial cells induces the peristaltic transport of sweat in human eccrine glands
Source: Commun Biol. 2023 Nov 18;6:1175. doi: 10.1038/s42003-023-05557-9 (PMC10657463; doi:10.1038/s42003-023-05557-9)
Supplement: Supplementary file 3 — Description of Additional Supplementary Files [file 42003_2023_5557_MOESM3_ESM.pdf]

Description of Additional Supplementary Files

File name: Supplementary Data 1
Description: The source data behind the graphs in the paper.

File name: Supplementary Movie 1
Description: Time-lapse imaging of an isolated eccrine gland costained to visualize actin (**middle**) and nuclei (**right**) without pilocarpine stimulation.

File name: Supplementary Movie 2
Description: Time-lapse imaging of an isolated eccrine gland costained to visualize actin (**middle**) and nuclei (**right**) after pilocarpine treatment.

File name: Supplementary Movie 3
Description: Original (**left**) and color aberration (**right**) time-lapse images of actin (**upper**) and nuclei (**lower**) after pilocarpine treatment in the KRS condition.

File name: Supplementary Movie 4
Description: Time-lapse imaging of an isolated eccrine gland after pilocarpine treatment without Acti-stain.

File name: Supplementary Movie 5
Description: High-speed recording of the contraction of a human eccrine gland.

File name: Supplementary Movie 6
Description: Original image of nuclear movement (**left**) and the corresponding color-aberration image with the starting point in green and another time point in magenta (**right**) after pilocarpine treatment (control).

File name: Supplementary Movie 7
Description: Inhibition of pilocarpine-induced contraction with atropine pretreatment. Original image of nuclear movement (**left**) and the corresponding color-aberration image with the starting point in green and another time point in magenta (**right**).

File name: Supplementary Movie 8

Description: Original image of nuclear movement (**left**) and the corresponding color-aberration image with the starting point in green and another time point in magenta (**right**) after nicotine treatment. No displacement along the Z-axis was observed. The magenta signals emerging in the left part of the duct were due to fluorescence quenching over time.

File name: Supplementary Movie 9

Description: Original and color aberration images of contractile motions of a whole CMDR-stained eccrine gland.

File name: Supplementary Movie 10

Description: High-magnification time-lapse images of the secretory duct with concertina CMDR signals (see also Fig. 6**a,b**).

File name: Supplementary Movie 11

Description: High-magnification time-lapse images in the secretory duct with thick stripe CMDR signals (see also Fig. 6**a,b**).

File name: Supplementary Movie 12

Description: Original (**left**) and color aberration (**right**) time-lapse images of nuclei in the secretory duct after pretreatment with cytochalasin D.

File name: Supplementary Movie 13

Description: Time-lapse images of a CMDR-stained eccrine gland focusing on the changes in the lumen area in the whole coiled portion after pilocarpine treatment. The lumen of the secretory duct is colored green, and those of the excretory duct are colored magenta and blue.

File name: Supplementary Movie 14

Description: Original (**left**) and color aberration (**right**) time-lapse images of nuclei in the secretory duct with pilocarpine stimulation and no pretreatment with CBX.

File name: Supplementary Movie 15

Description: Original (**left**) and color aberration (**right**) time-lapse images of nuclei in the secretory duct pretreated with CBX before pilocarpine stimulation.

File name: Supplementary Movie 16
Description: Original (**left**) and color aberration (**right**) time-lapse images of nuclei in the secretory duct pretreated with 2-APB before pilocarpine stimulation.

File name: Supplementary Movie 17
Description: Original (**left**) and color aberration (**right**) time-lapse images of nuclei in the secretory duct pretreated with GAP27 before pilocarpine stimulation.
