# Supplementary material for: Gap junction-mediated contraction of myoepithelial cells induces the peristaltic transport of sweat in human eccrine glands
Source: Commun Biol. 2023 Nov 18;6:1175. doi: 10.1038/s42003-023-05557-9 (PMC10657463; doi:10.1038/s42003-023-05557-9)
Supplement: Supplementary file 22 — Reporting Summary [file 42003_2023_5557_MOESM22_ESM.pdf]

nature portfolio

Corresponding author(s): Kiyotoshi Sekiguchi

Last updated by author(s): Oct 28, 2023

Reporting Summary

Nature Portfolio wishes to improve the reproducibility of the work that we publish. This form provides structure for consistency and transparency in reporting. For further information on Nature Portfolio policies, see our Editorial Policies and the Editorial Policy Checklist.

Statistics

For all statistical analyses, confirm that the following items are present in the figure legend, table legend, main text, or Methods section.

n/a	Confirmed	
☐	☒	The exact sample size (*n*) for each experimental group/condition, given as a discrete number and unit of measurement
☐	☒	A statement on whether measurements were taken from distinct samples or whether the same sample was measured repeatedly
☐	☒	The statistical test(s) used AND whether they are one- or two-sided<br>*Only common tests should be described solely by name; describe more complex techniques in the Methods section.*
☐	☒	A description of all covariates tested
☒	☐	A description of any assumptions or corrections, such as tests of normality and adjustment for multiple comparisons
☐	☒	A full description of the statistical parameters including central tendency (e.g. means) or other basic estimates (e.g. regression coefficient) AND variation (e.g. standard deviation) or associated estimates of uncertainty (e.g. confidence intervals)
☐	☒	For null hypothesis testing, the test statistic (e.g. *F*, *t*, *r*) with confidence intervals, effect sizes, degrees of freedom and *P* value noted<br>*Give P values as exact values whenever suitable.*
☒	☐	For Bayesian analysis, information on the choice of priors and Markov chain Monte Carlo settings
☒	☐	For hierarchical and complex designs, identification of the appropriate level for tests and full reporting of outcomes
☒	☐	Estimates of effect sizes (e.g. Cohen's *d*, Pearson's *r*), indicating how they were calculated

Our web collection on statistics for biologists contains articles on many of the points above.

Software and code

Policy information about availability of computer code

Data collection	Fluoview 1200, iQ3, Imaris Ver.8.2.1, ViiA7 real-time PCR system, FACSAria2
Data analysis	Excel 2019, Kaleidagraph Ver.4.5, Imaris Ver.8.2.1, ImageJ, FACSAria2

For manuscripts utilizing custom algorithms or software that are central to the research but not yet described in published literature, software must be made available to editors and reviewers. We strongly encourage code deposition in a community repository (e.g. GitHub). See the Nature Portfolio guidelines for submitting code & software for further information.

Data

Policy information about availability of data

All manuscripts must include a data availability statement. This statement should provide the following information, where applicable:

- Accession codes, unique identifiers, or web links for publicly available datasets
- A description of any restrictions on data availability
- For clinical datasets or third party data, please ensure that the statement adheres to our policy

All relevant data are provided in the manuscript along with the supplementary materials or are available from the authors upon request.

Field-specific reporting

Please select the one below that is the best fit for your research. If you are not sure, read the appropriate sections before making your selection.

☒ Life sciences ☐ Behavioural & social sciences ☐ Ecological, evolutionary & environmental sciences

For a reference copy of the document with all sections, see nature.com/documents/nr-reporting-summary-flat.pdf

Life sciences study design

All studies must disclose on these points even when the disclosure is negative.

Sample size	For live imaging, 8 sweat glands from 4 people were observed and a minimum 5 (5-14) of nuclei movement were measured.
Data exclusions	No data were excluded from the present study.
Replication	All the experiments were shown with the number of replicates (independent nuclei, samples, and participants are shown by n) with SD for all the experiments where appropriate.
Randomization	The nuclei of the same field were randomly chosen for measurement.
Blinding	In most of the experiments, no blinding method was possible as only one experimenter was performing the analysis.

Reporting for specific materials, systems and methods

We require information from authors about some types of materials, experimental systems and methods used in many studies. Here, indicate whether each material, system or method listed is relevant to your study. If you are not sure if a list item applies to your research, read the appropriate section before selecting a response.

Materials & experimental systems

n/a	Involved in the study	
☐	☒	Antibodies
☒	☐	Eukaryotic cell lines
☒	☐	Palaeontology and archaeology
☒	☐	Animals and other organisms
☐	☒	Human research participants
☒	☐	Clinical data
☒	☐	Dual use research of concern

Methods

n/a	Involved in the study	
☒	☐	ChIP-seq
☐	☒	Flow cytometry
☒	☐	MRI-based neuroimaging

Antibodies

Antibodies used

Rabbit anti-alpha smooth muscle actin antibody (Lot# GR3183259-35; ab5694; Abcam, Cambridge, UK)
Mouse anti-PGP9.5 antibody (Lot# GR241581-3; ab8189; Abcam, Cambridge, UK)
Alexa Fluor 594 conjugated secondary antibodies (Lot# 2160431; A11037; Thermo Fisher Scientific)
allophycocyanin-conjugated anti-CD29 (1:6, 559883, BD Pharmingen, San Diego, CA)
Brilliant Violet 421-conjugated anti-CD49f (1:20, 313624, BioLegend, San Diego, CA)
anti-Cx26 (1:500, ab59020, Abcam, Cambridge, UK), anti-Cx30 (1:100, HPA014846, Sigma–Aldrich), anti-Cx31 (1:50, LS-C375784, LSBio, WA, USA), anti-Cx37 (1:125, #40-4300, Invitrogen), anti-Cx43 (1:1000, ab11370, Abcam), anti-Cx45 (1:100, CX45B12-A, Alpha Diagnostics) and anti-αSMA (1:100, A2547, Merck)
Alexa Fluor 488-conjugated goat anti-mouse IgG, and Alexa Fluor 594-conjugated donkey anti-rabbit or anti-goat IgG (1:200, A21202, A21207, A11058, Thermo Fisher)

Validation

The antibodies used in this study were validated with positive controls in eccrine glands (Yamaga et al., 2018, JID) or in our previous study (Kurata et al., 2017, PlosOne).
Anti-Cx series antibodies are validated in the following papers:
anti-Cx26:Fort AG, Murray JW, Dandachi N, Davidson MW, Dermietzel R, Wolkoff AW, Spray DC. In vitro motility of liver connexin vesicles along microtubules utilizes kinesin motors. J Biol Chem. 2011 Jul 1;286(26):22875-85. doi: 10.1074/jbc.M111.219709. Epub 2011 May 2. PMID: 21536677; PMCID: PMC3123055.
anti-Cx30:Hosoya, M., Fujioka, M., Ogawa, K. et al. Distinct Expression Patterns Of Causative Genes Responsible For Hereditary Progressive Hearing Loss In Non-Human Primate Cochlea. Sci Rep 6, 22250 (2016). https://doi.org/10.1038/srep22250
anti-Cx31:MrgX2 is a high potency cortistatin receptor expressed in dorsal root ganglion. Robas N, Mead E, Fidock M. The Journal of biological chemistry. 2003 278:44400-4. [PubMed:12915402]
anti-Cx37:El-Hayek S, Clarke HJ. Follicle-Stimulating Hormone Increases Gap Junctional Communication Between Somatic and Germ-Line Follicular Compartments During Murine Oogenesis. Biol Reprod. 2015 Aug;93(2):47. doi: 10.1095/biolreprod.115.129569. Epub 2015 Jun 10. PMID: 26063870.

anti-Cx43:Potter JA, Price GW, Cliff CL, Green CR, Squires PE, Hills CE. Collagen I Modifies Connexin-43 Hemichannel Activity via Integrin α2β1 Binding in TGFβ1-Evoked Renal Tubular Epithelial Cells. Int J Mol Sci. 2021 Mar 31;22(7):3644. doi: 10.3390/ijms22073644. PMID: 33807408; PMCID: PMC8038016. etc.
anti-Cx45: Wei-Li Di, Elizabeth L. Rugg, Irene M. Leigh, David P. Kelsell, Multiple Epidermal Connexins are Expressed in Different Keratinocyte Subpopulations Including Connexin 31, Journal of Investigative Dermatology,Volume 117, Issue 4,2001,https://doi.org/10.1046/j.0022-202x.2001.01468.x.

Human research participants

Policy information about studies involving human research participants

Population characteristics

Six healthy participants include two Asian females and four Asian males. The range of participants' age was from 24 to 41 with an average of 31.

Recruitment

Volunteers were recruited from healthy young adults with informed consent.

Ethics oversight

Active sweating vivo test was approved by the Ethics Committees of Mandom Corporation (Osaka, Japan).
Experiments using the human skin samples were approved by the Ethics Committees of Osaka University (Yakukumi 28-3, Yakukumi 28-5, Yakukumi 29-2, Yakujin-2019-10). Measurements of active sweating in vivo were approved by the Ethics Committees of Mandom Corporation (Osaka, Japan).

Note that full information on the approval of the study protocol must also be provided in the manuscript.

Flow Cytometry

Plots

Confirm that:

☒ The axis labels state the marker and fluorochrome used (e.g. CD4-FITC).

☒ The axis scales are clearly visible. Include numbers along axes only for bottom left plot of group (a 'group' is an analysis of identical markers).

☒ All plots are contour plots with outliers or pseudocolor plots.

☒ A numerical value for number of cells or percentage (with statistics) is provided.

Methodology

Sample preparation

Fresh skin tissues were minced and enzymatically disaggregated for 16 hours at 37°C in Complete MammoCult Human medium (Stem Cell Technologies, Vancouver, BC, Canada) with 600 U/ml collagenase type II (Worthington Biochemicals, Freehold, NJ, USA) on a tube rotator. After centrifugation at 300×g for 5 minutes, a single-cell suspension was obtained by repeated pipetting for 3 minutes in prewarmed 0.5% trypsin/1 mM EDTA in PBS, followed by repeated pipetting for 1 minute in prewarmed 5 mg/ml dispase (Gibco, Paisley, UK)/0.1 mg/ml DNase I (Sigma). The resulting suspension was filtered through a 40-µm mesh (BD Biosciences, San Jose, CA, USA). Immunolabeling of cells was performed at 4°C for 30 minutes in PBS containing 5% BSA with allophycocyanin-conjugated anti-CD29 (1:6, 559883, BD Pharmingen, San Diego, CA) and Brilliant Violet 421-conjugated anti-CD49f (1:20, 313624, BioLegend, San Diego, CA). After being washed twice with 5% BSA in PBS, the cells were resuspended in PBS with 7-AAD (1:100, BioLegend) for dead cell staining. The dead cells were excluded from subsequent analyses.

Instrument

FACSAria2 (BD Biosciences) flow cytometer

Software

FACSAria2

Cell population abundance

In all experiments, an unstained control sample lacking a primary antibody was used and at least 10,000 events were recorded. CD29high CD49fhigh population was 20-25% in the live cell population.

Gating strategy

7AAD stained control was used as a negative control for gating of CD29 and CD49 positive population.

☐ Tick this box to confirm that a figure exemplifying the gating strategy is provided in the Supplementary Information.
